# Influence of calcium on ceramide-1-phosphate monolayers

**DOI:** 10.3762/bjnano.7.22

**Published:** 2016-02-12

**Authors:** Joana S L Oliveira, Gerald Brezesinski, Alexandra Hill, Arne Gericke

**Affiliations:** 1 Max Planck Institute of Colloids and Interfaces, Colloid Chemistry Department, Wissenschaftspark Potsdam-Golm, Am Mühlenberg 1, 14476 Potsdam, Germany; 2 Department of Biological Sciences, Kent State University, Kent, Ohio 44242, USA

**Keywords:** calcium, ceramide-1-phosphate, Langmuir monolayers, phase behaviour, structural properties

## Abstract

Ceramide-1-phosphate (C1P) plays an important role in several biological processes, being identified as a key regulator of many protein functions. For instance, it acts as a mediator of inflammatory responses. The mediation of the inflammation process happens due to the interaction of C1P with the C2 domain of cPLA_2α_, an effector protein that needs the presence of submicromolar concentrations of calcium ions. The aim of this study was to determine the phase behaviour and structural properties of C1P in the presence and absence of millimolar quantities of calcium in a well-defined pH environment. For that purpose, we used monomolecular films of C1P at the soft air/liquid interface with calcium ions in the subphase. The pH was varied to change the protonation degree of the C1P head group. We used surface pressure versus molecular area isotherms coupled with other monolayer techniques as Brewster angle microscopy (BAM), infrared reflection–absorption spectroscopy (IRRAS) and grazing incidence X-ray diffraction (GIXD). The isotherms indicate that C1P monolayers are in a condensed state in the presence of calcium ions, regardless of the pH. At higher pH without calcium ions, the monolayer is in a liquid-expanded state due to repulsion between the negatively charged phosphate groups of the C1P molecules. When divalent calcium ions are added, they are able to bridge the highly charged phosphate groups, enhancing the regular arrangement of the head groups. Similar solidification of the monolayer structure can be seen in the presence of a 150 times larger concentration of monovalent sodium ions. Therefore, calcium ions have clearly a strong affinity for the phosphomonoester of C1P.

## Introduction

**Figure 1:**
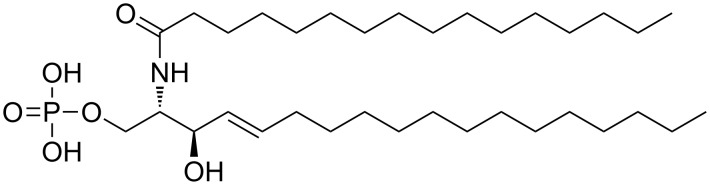
Chemical structure of fully protonated C1P.

Ceramide-1-phosphate (C1P) is a sphingoid analogue of phosphatidic acid, which has a sphingoid base with a phosphomonoester head group (Figure 1). It is synthesized in the trans-Golgi network (TGN), where ceramide is phosphorylated into C1P in a reaction catalyzed by a ceramide kinase [1–2]. Several reviews about the function of C1P describe its role in a number of biological functions as cell growth, survival and mediation of macrophage migration and control of inflammatory responses [3–5].

According to the work of Chalfant and co-workers [6–7], C1P mediates inflammatory responses by activation of the cytosolic phospholipase A2α (cPLA_2_α). cPLA_2_α is the major phospholipase that regulates eicosanoid synthesis in response to inflammatory agonists. The binding site for C1P was determined to be within the C2-domain of cPLA_2_α, by a calcium-dependent mechanism [3,6–7].

For a better understanding of the mechanisms underlying the described biological functions, Langmuir monolayers at the soft air/water interface are convenient model systems. They can be especially used to study interactions at membrane surfaces. By changing the subphase conditions, such as pH, ionic strength, or/and the type of ions (Hofmeister effects), it is possible to study their influence on the phase state of the lipids. Having this in mind, Kooijman and co-workers [8] studied the behaviour of C1P monolayers in the absence and presence of Ca^2+^ and found interesting results. On pure water, C1P forms a condensed monolayer, while at pH 7.2 the electrostatic repulsion of the negatively charged phosphomonoester head groups becomes more important and the monolayer is in a less solid-like state. Also at pH 7.2, the monolayer has a higher charge density than on water. They also determined the p*K*_a2_ in presence of other lipids (DOPC and DOPE), and found that the environment has a strong influence on the charge state of the phosphomonoester head groups [9]. The p*K*_a2_ of C1P is in the range of physiological values (5 < pH < 8) and similar to the one of lyso-phosphatidic acid (LPA) [10].

Since the monolayer studies were performed in a non-defined protonation region, either water or pH 7.2, we studied the phase behaviour and structural properties at more extreme values, namely at pH 4, where C1P should be mostly protonated, and at pH 9, where we expect the C1P to be completely deprotonated. The aim of these experiments is to provide a complete physicochemical characterization of C1P towards a better understanding of the influence of pH and ions in the subphase. This study can lead to better comprehension of the mechanism and potential role of this important sphingolipid in biological membranes and cellular environment.

## Results and Discussion

**Figure 2:**
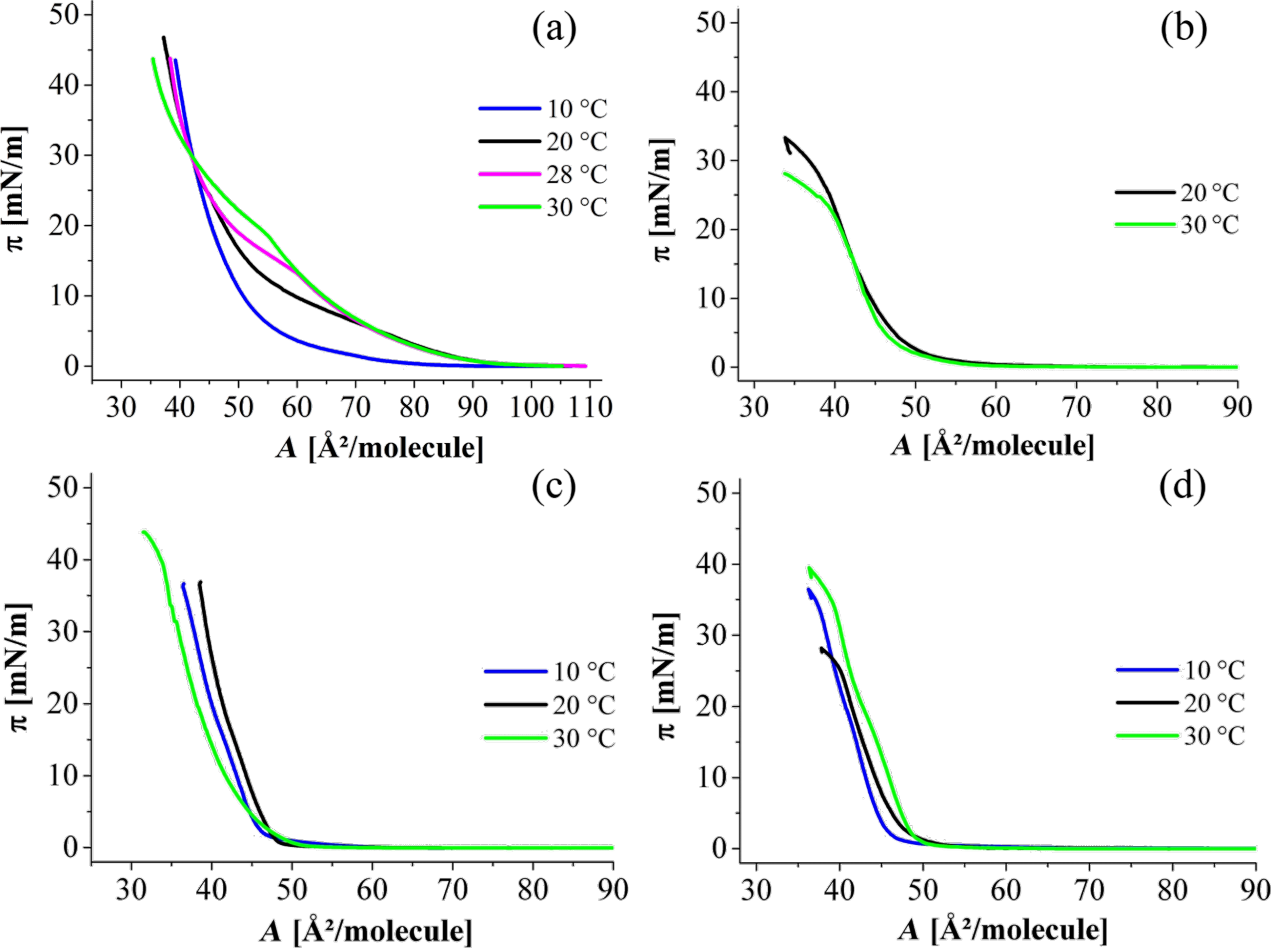
Surface pressure–area isotherms at (a) pH 9 without calcium (borax buffer with 150 mM NaCl and 1 mM EDTA), (b) pH 9 with calcium (borax buffer with 1 mM CaCl_2_ and 150 mM NaCl), (c) pH 4 without calcium (citric buffer with 150 mM NaCl and 1 mM EDTA), and (d) pH 4 with calcium (citric buffer with 1 mM CaCl_2_ and 150 mM NaCl) at different temperatures (indicated).

The C1P monolayer was studied at pH 4 and pH 9. While at pH 9 the head groups of C1P should be mostly deprotonated and therefore twofold negatively charged, these groups are highly protonated at pH 4. Figure 2 displays the surface pressure–area isotherms at pH 4 and pH 9 with and without calcium in the subphase.

For pH 9, a solution of 10 mM borax buffer has been used, and for pH 4, a 10 mM citric buffer. Either 150 mM NaCl was added to mimic the physiological environment and 1 mM EDTA to chelate traces of divalent cations, or the EDTA was substituted by 1 mM CaCl_2_.

On the pH 9 subphase without calcium, the isotherms indicate an expanded phase (LE) at low lateral pressures and a first-order phase transition from LE to LC (condensed state). At 10 °C, this phase transition is not seen. As the temperature increases, the phase-transition pressure increases. This dependence is characteristic of a first-order transition. Unfortunately, the domains appearing in the two-phase coexistence region are too small for the lateral resolution of our BAM instrument (about 2 µm), and therefore we were not able to follow the nucleation and growth processes associated with the LE/LC phase transition (data not shown). The calculated critical temperature *T*_c_ (see section 1 in Supporting Information File 1) for this system, i.e., the temperature above which the LC phase cannot be reached anymore upon compression of the monolayer, was found to be about 38 °C, which is slightly lower than the transition temperature *T*_m_ of a gel to a liquid-crystalline phase obtained by Kooijman et al. [9] (ca. 44 °C) for the bulk system at pH 7.4. Most likely, the difference in temperatures can be attributed to a different protonation state of C1P in the two cases. Therefore, we might conclude that *T*_c_ and *T*_m_ are the same showing that the lateral packing in the monolayers and in the bilayers is identical [11].

At pH 4 without calcium, the isotherms have a shape characteristic of a condensed phase. The resublimation (transition from gas-analogous to condensed state) occurs at nearly zero pressure. As a first assumption, we expect the C1P head groups to be mostly deprotonated at pH 9. This leads to strong electrostatic repulsion between the twofold negatively charged phosphomonoester head groups favouring the disordered LE phase. In contrast, the head groups are expected to be mostly protonated at pH 4 allowing the molecules to pack in a tighter way, favouring the ordered LC phase.

**Figure 3:**
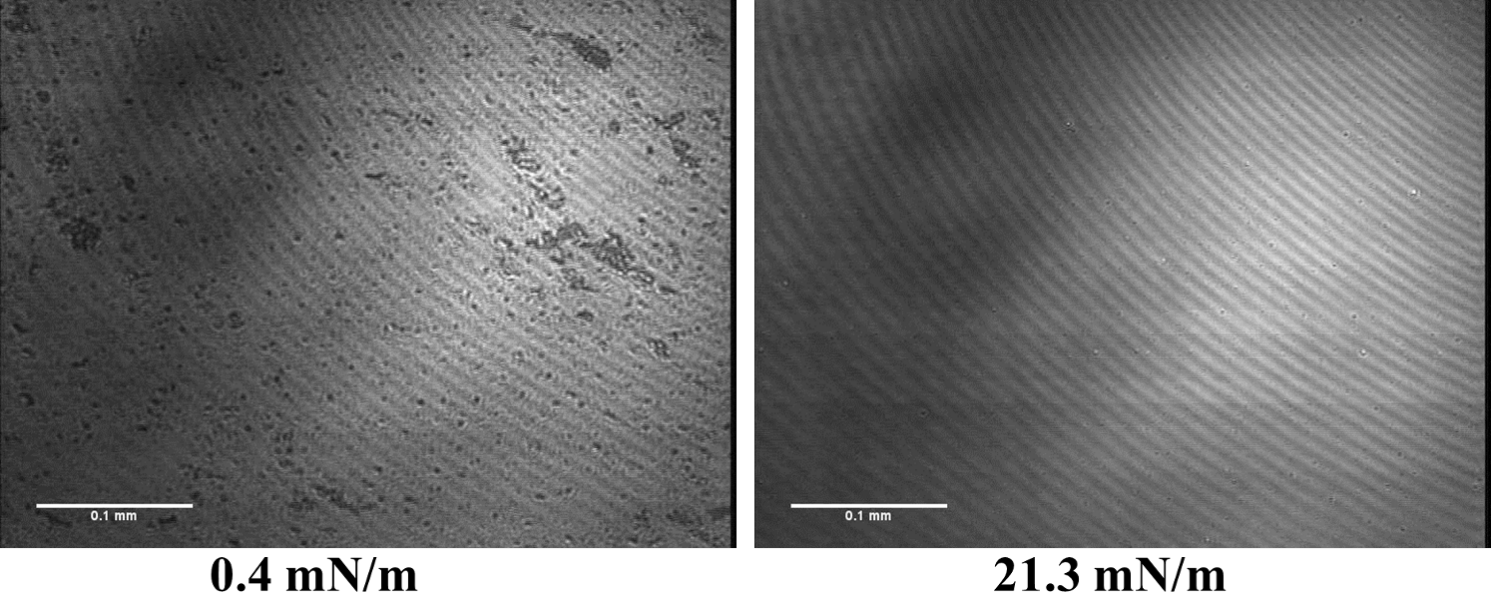
BAM pictures obtained at pH 4 without calcium (citric buffer with 150 mM NaCl and 1 mM EDTA) at selected surface pressures (indicated).

When calcium is added, the isotherms of the pH 9 subphase change drastically. Now, the typical behaviour of a fully condensed monolayer even at low pressures can be seen. At pH 4, no pronounced changes are seen, and the monolayer is at all lateral pressures in the condensed state. The isotherm on water is also fully condensed (Figure S1, Supporting Information File 1), which is in perfect agreement with the isotherm obtained by Kooijman [8]. This is not surprising, since the pH of water saturated with CO_2_ is around 6. All the condensed isotherms are supported by Brewster angle microscopy images. Figure 3 is shown as an example of a stiff and crystalline monolayer observed on a pH 4 subphase without calcium. At very low pressure, the image indicates some holes, which are removed by compression.

The monolayers in the condensed state are not perfectly reproducible concerning the areas per molecule (as already reported by Kooijman [8]). They exhibit hysteresis between compression and decompression, which proves the highly crystalline state of these systems and leads to limitations in a precise determination of molecular areas. The isotherms were measured at several temperatures to investigate whether the first-order transition between LE and LC can be obtained. However, only condensed monolayers have been obtained in the accessible temperature range. Only at pH 4 without calcium, the isotherm measured at 30 °C is clearly shifted to smaller area values, most probably due to a slightly increased solubility of C1P in this subphase.

**Figure 4:**
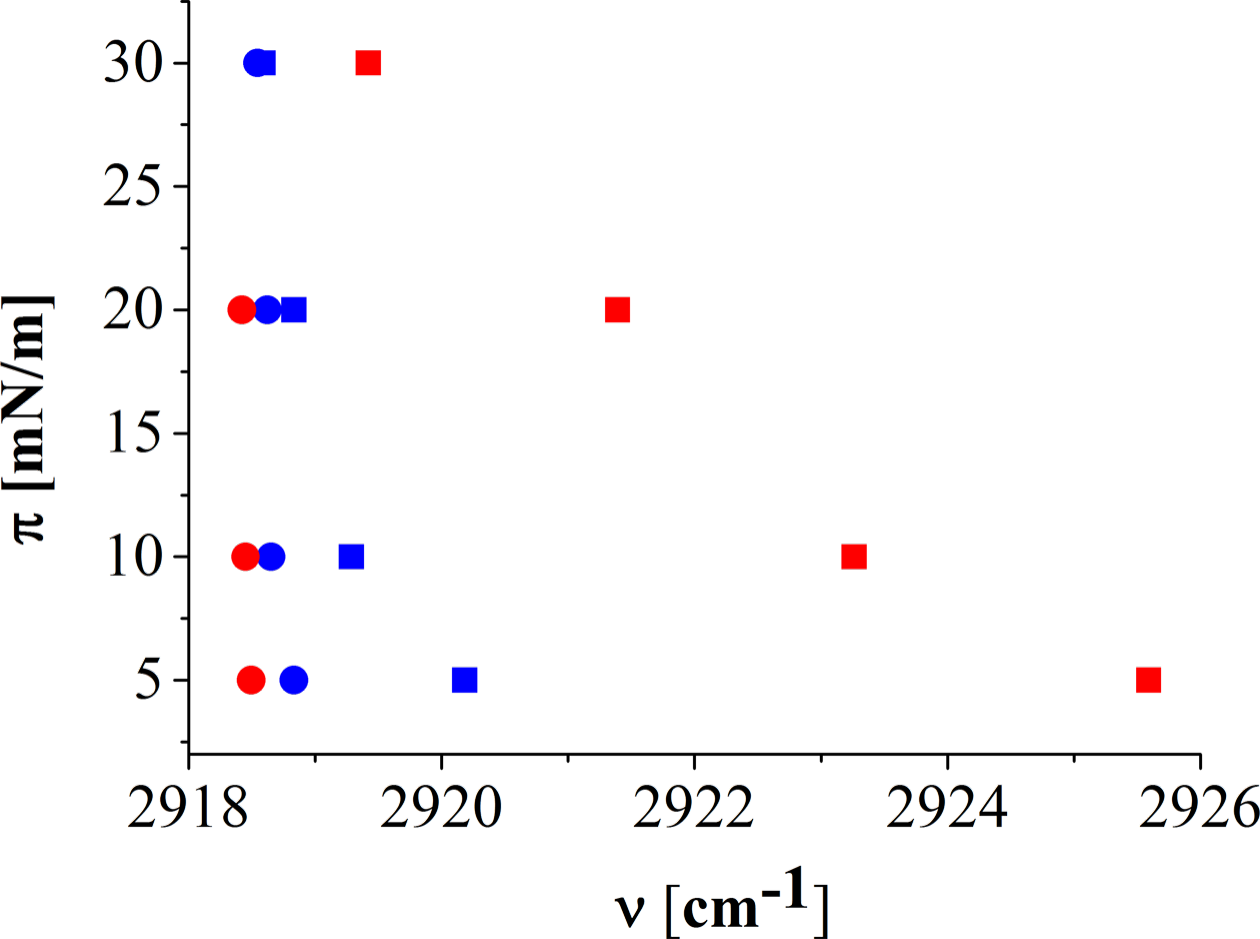
Wavenumbers of the CH_2_ asymmetric stretching vibration band (incidence angle 40° and s-polarization) of C1P monolayers at 30 °C on different subphases: (red square) pH 9 without calcium (borax buffer with 150 mM NaCl and 1 mM EDTA), (red circles) pH 9 with calcium (borax buffer with 1 mM Ca^2+^ and 150 mM NaCl), (blue squares) pH 4 without calcium (citric buffer with 150 mM NaCl and 1 mM EDTA), and (blue circle) pH 4 with calcium (citric buffer with 1 mM Ca^2+^ and 150 mM NaCl), determined by IRRAS.

Infrared reflection–absorption spectroscopy (IRRAS) has been used to determine the phase state of the aliphatic chains on the different subphases. For this purpose, the CH_2_ symmetric and asymmetric stretching vibration bands of IRRAS spectra have been carefully analyzed. If the chains are in an ordered condensed (all-*trans*) state, the corresponding characteristic bands are at wavenumbers ν ≤ 2920 cm^−1^ (ν_asym_CH_2_) and ν ≤ 2849 cm^−1^ (ν_sym_CH_2_). Wavenumbers ν ≥ 2924 cm^−1^ (ν_asym_CH_2_) or ν ≥ 2854 cm^−1^ (ν_sym_CH_2_) are characteristic for the liquid-like (*gauche*) state of the chains [12]. Figure 4 displays the wavenumbers of the ν_asym_CH_2_ band of C1P on the different subphases.

The IRRAS data show the same tendency as the isotherms. At pH 4 without calcium, C1P is in a condensed state with slightly higher wavenumbers at low pressures (less dense packing of molecules). Upon compression, the same low wavenumbers (approx. 2918.5 cm^−1^) are reached as in the system with calcium (the packing and therefore the wavenumbers do not change upon compression). At pH 9 without calcium, high wavenumbers at low pressures (approx. 2926 cm^−1^) indicate the disordered LE state, which upon compression undergoes the transition into the LC phase with lower wavenumbers (approx. 2920 cm^−1^), but not as low as in the other systems. At pH 9, the electrostatic repulsion between the negatively charged head groups leads to an expanded state. These repulsive forces prevent a high packing density even at high lateral pressures. Interestingly, on the pH 9 subphase with calcium, the C1P chains are in a highly condensed state (all-*trans* conformation) with very low wavenumbers (approx. 2918.2 cm^−1^). All temperatures studied show the same tendency (data of IRRAS measurements at 10 °C and 30 °C not shown).

**Figure 5:**
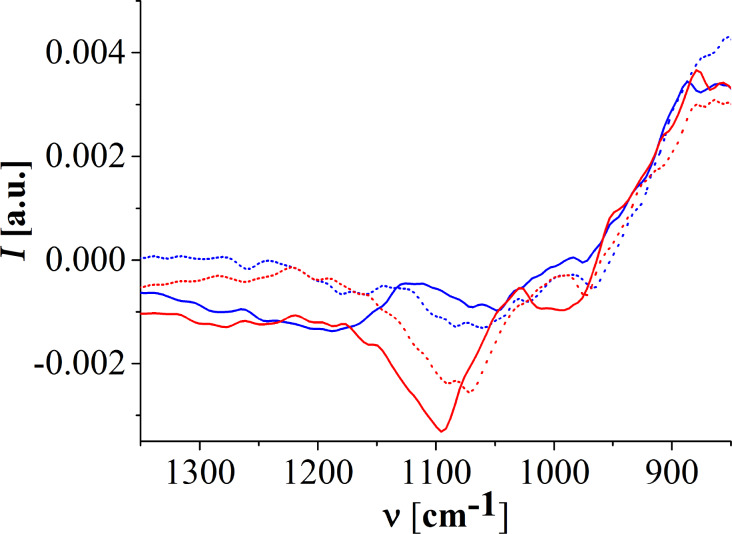
IRRAS spectra (incidence angle 40°, s-polarization) of C1P monolayers on the different subphases (red lines – pH 9 buffer; blue lines – pH 4 buffer; solid line – with calcium; dotted line – without calcium) at 20 mN/m and 30 °C. The spectra are shifted (*y*-axis) for better clarity.

C1P has three dissociation states due to its phosphomonoester head group. The equilibrium constants p*K*_a1_ and p*K*_a2_ determine the state of C1P, defining whether the molecule will be completely deprotonated, one-fold deprotonated or neutral (no deprotonation) at a certain pH. The p*K*_a2_ of C1P was determined by Kooijman et al. [9] to lie within pH 5 and 8. The dissociation behaviour of C1P was shown to be similar to the one of LPA, obeying the electrostatic/hydrogen bond switch model, and to a certain extent to the one of phosphatidic acid (PA) [10]. The p*K*_a1_ of LPA and PA is in the pH range between 2 and 4 [13]. To our knowledge, the p*K*_a1_ of C1P is not described in the literature. We expect C1P to be one-fold deprotonated at pH 4 and completely deprotonated at pH 9. In an attempt to get more information about the deprotonation state of the C1P monolayer, the phosphate vibration bands of IRRAS spectra were analyzed and are depicted in Figure 5. The asymmetrical and symmetrical stretching vibration bands of PO_3_^2−^ are described to be located at approx. 1080 cm^−1^ and approx. 1000 cm^−1^ and the P–O–(H) stretching mode at approx. 925 cm^−1^ [14].

It is important to note that the phosphate bands region is noisy and the accurate analysis of the peak position of the bands is challenging. Therefore no fitting was performed, and the peak positions were defined by an eye analysis. Apart from the asymmetrical PO_3_^2−^ band of C1P on the pH 9 subphase with calcium, which shows a pronounced intensity, the bands are either very broad or seem to be a doublet. Doublets were already described for phosphonic acid salts in IR [14]. However, the broadening of the bands could also indicate a superposition of bands due to different protonation states of the molecules in the system.

At pH 4 without calcium, a band at approx. 970 cm^−1^ and a very broad band composed of at least two contributions, one doublet around 1012 cm^−1^ and the other one around 1068 cm^−1^ are present. In comparison with LPA and PA, the p*K*_a1_ of C1P is expected to be in the range of 2 < pH < 4, hence at pH 4 C1P should be one-fold deprotonated. The ionic strength of the subphase can influence the dissociation degree of the monolayer, since the counter-ions interact with the charged head groups. Usually the p*K*_a_ values decrease with increasing ionic strength. The doublets seem to indicate a coexistence of one-fold deprotonated and neutral C1Ps. When calcium is added to the system such bands change slightly the position to approx. 966 cm^−1^, approx. 1007 cm^−1^ (very weak band) and one doublet around 1066 cm^−1^ (approx. 1052 and approx. 1079 cm^−1^). The slightly lower wavenumbers and shape of the bands give an overall impression that almost all C1P molecules are now neutral. The wavenumbers of the acyl chains support this assumption: the monolayer without calcium has slightly lower wavenumbers (less condensed monolayer) due to electrostatic repulsions between the charged oxygen ions of the phosphomonoester head group. Interestingly, molecular dynamics simulations of dimyristoylphosphatidate (DMPA^−^) monolayer in water environment (and therefore one-fold deprotonated) reported no difference between the effect of Ca^2+^ or Na^+^ ions on the properties of a condensed phase monolayer [15].

At pH 9, C1P is expected to be completely deprotonated. The wavenumbers are shifted to higher values. The bands for the subphase without calcium are located at approx. 974 cm^−1^, approx. 1011 cm^−1^ and a doublet (approx. 1072 and 1088 cm^−1^). We assume C1P on the pH 9 subphase without calcium is most likely not 100% deprotonated since the doublet is still present. When calcium is added to the subphase the wavenumbers are even more shifted to higher wavenumbers and the asymmetrical PO_3_^2−^ band is no longer a doublet. The bands are located at approx. 995 cm^−1^, approx. 1045 cm^−1^ and 1100 cm^−1^. Such clear shift to higher wavenumbers was already observed by Laroche et al. [14] in the case of a DMPA/Ca^2+^ complex, where the bridging of the head groups of neighbouring molecules mediated by calcium was found. Nevertheless, even if the phosphate bands indicate that Ca^2+^ ions have a stronger influence on the charge state of the monolayer (slightly deprotonated at pH 9) compared to a higher concentration of Na^+^, no quantitative information about the deprotonation state of the monolayer is available. Again, at all temperatures and pressures studied the same tendency was found.

This interesting calcium dependence was further investigated by means of grazing incidence X-ray diffraction (GIXD) at synchrotron facilities. The Langmuir trough is in an air-tight container filled with helium to avoid signal reduction due to absorption and scattering of photons in air. The incident beam is at an angle below the critical angle for total external reflection. The diffraction pattern is then collected by a linear detector which is scanned in the in-plane direction. The parameters of the unit cell of the lipid chains can be calculated based on the *Q_xy_* and *Q_z_* values of the observed Bragg peaks. All the details of GIXD measurements and analysis can be found in [16–18].

**Figure 6:**
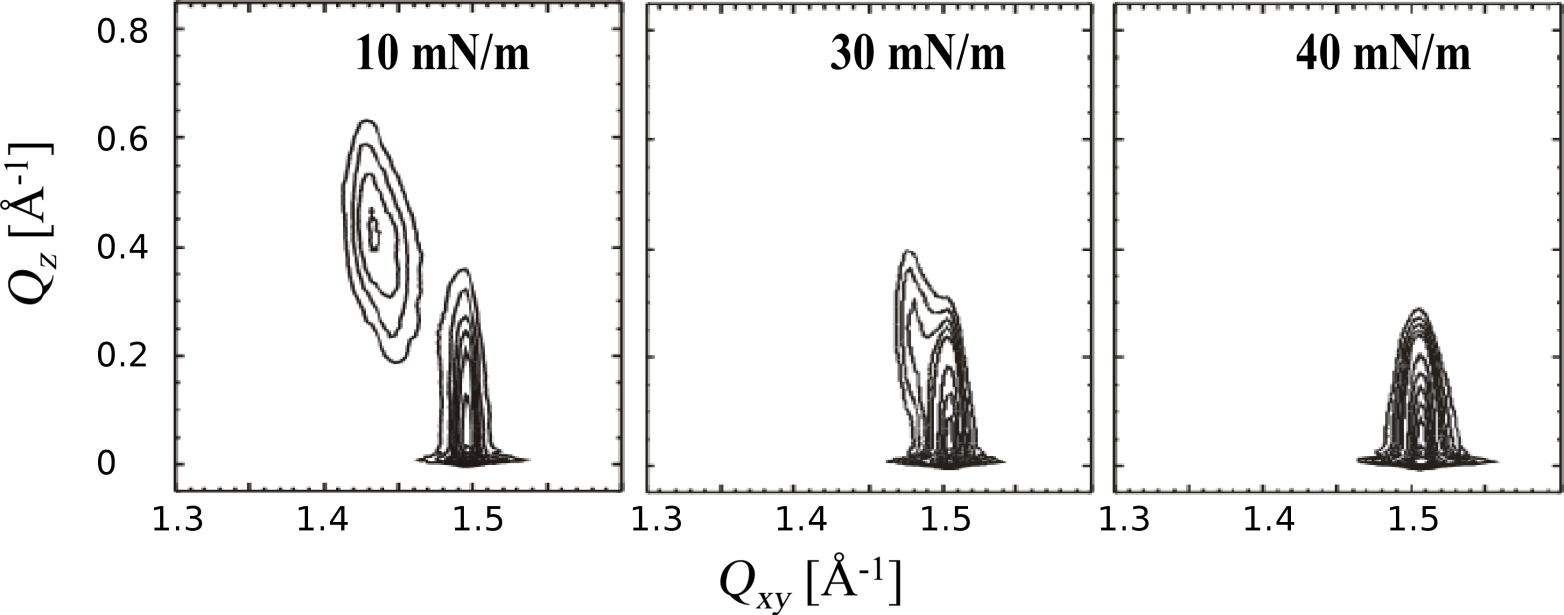
Contour plots of the corrected X-ray intensities plotted versus the in-plane (*Q_xy_*) and out-of-plane (*Q_z_*) components of the scattering vector for C1P monolayers on the pH 4 subphase with calcium (citric buffer with 1 mM Ca^2+^ and 150 mM NaCl) at 10 mN/m (left), 30 mN/m (middle) and 40 mN/m (right).

Figure 6 is shown as an example of the results obtained for C1P at pH 4 with calcium in the subphase. The contour plots display the diffracted intensities versus the in-plane (*Q_xy_*) and out-of-plane (*Q_z_*) components of the scattering vector. Bragg peaks and Bragg rods can be extracted to yield structural information as the in-plane lattice, the tilt of the chains, the lattice distortion, and the cross sectional area (*A*_0_) of one chain [16–18].

C1P on the pH 4 buffer at 10 mN/m shows three diffraction peaks indicative of the presence of an oblique in-plane lattice with a tilt of (18.6 ± 1.0)° and an area per molecule of (40.2 ± 0.8) Å^2^, which transforms between 10 and 30 mN/m into a rectangular lattice of NN (nearest neighbour) tilted chains. At 40 mN/m, only one Bragg peak has been observed indicating a hexagonal packing of untilted chains. The extrapolated tilting transition pressure is 38.9 mN/m (see below and section 3 in Supporting Information File 1).

On water, the presence of two Bragg peaks at non-zero *Q_z_* values demonstrates the existence of a rectangular unit cell with molecules tilted in the direction of next-nearest neighbours (NNN) and an area per molecule of (41.0 ± 0.8) Å^2^. Above 5 mN/m, the super-liquid (LS) phase (hexagonal unit cell with non-tilted molecules) is observed. Interestingly, the results obtained by Kooijman et al. [8] show a different picture, since at 10 mN/m the tilt angle is (18 ± 1)°. In our experiments, at the same pressure, non-tilted molecules are observed. This could be attributed to possible traces of divalent ions in the Millipore water used in our experiments with no addition of EDTA, and supports the finding that small amounts of divalent ions have a big influence on the behaviour of C1P.

**Figure 7:**
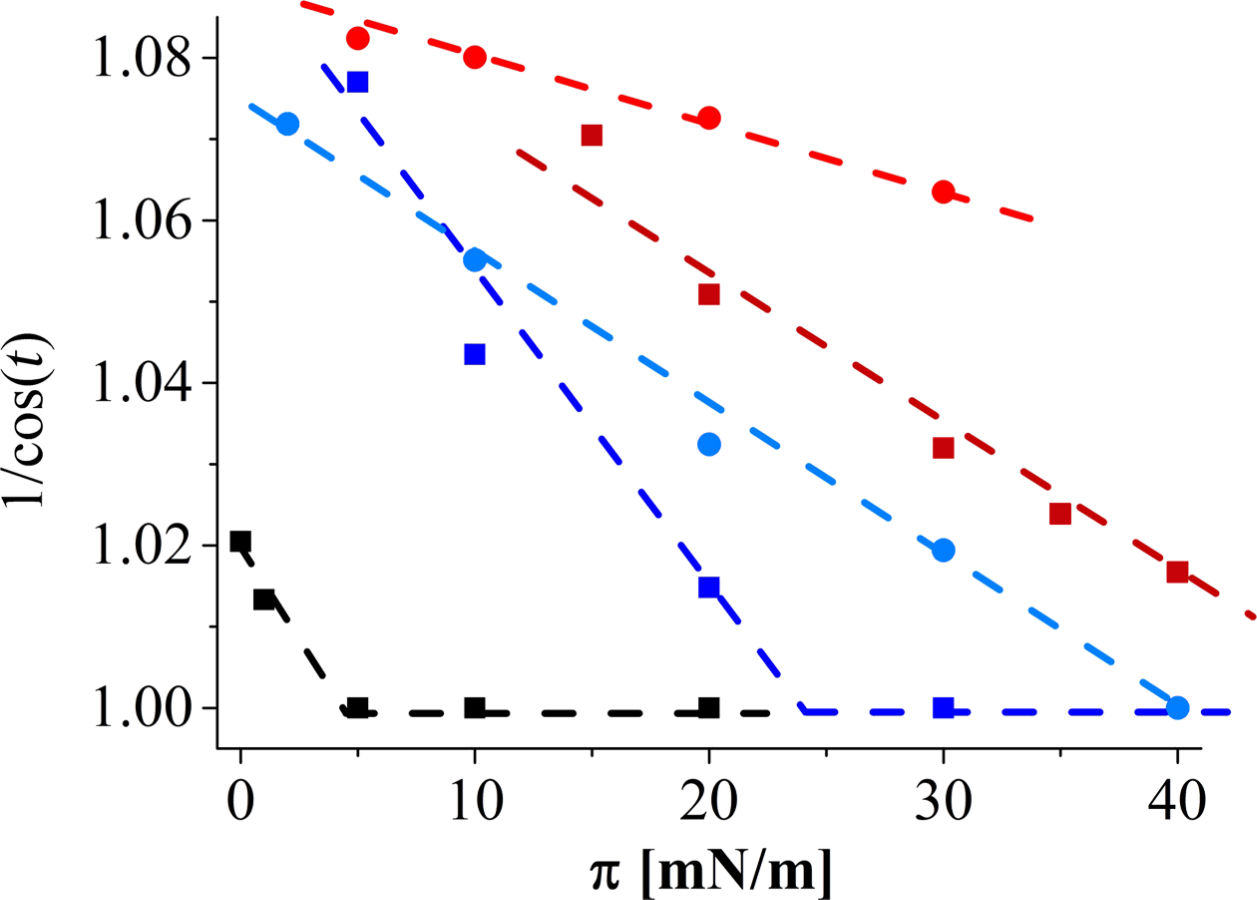
1/cos(*t*) as a function of the surface pressure of C1P monolayers at 20 °C on (red circle) pH 9 with calcium; (red square) pH 9 without calcium; (blue circle) pH 4 with calcium; (blue square) pH 4 without calcium; and (black square) on water.

Plotting 1/cos(*t*) versus the surface pressure (*t* is the tilt angle of the chains) allows for the determination of the tilting transition pressure. The only assumption one has to make is that the cross-sectional area of the chains remains constant on compression and that the isotherms show an almost linear relationship between area and pressure in the condensed state [19]. In Figure 7, the lines are linear fits to the experimental data. On water, the tilting transition occurs already at low lateral pressures (5.2 mN/m). As already discussed, at pH 4 with calcium the extrapolated tilting transition pressure is 38.9 mN/m.

At the same pH but without calcium in the subphase, the chains are packed in an oblique in-plane lattice at 5 mN/m, but with a larger tilt angle of (21.8 ± 1)°. Consequently, the in-plane area per molecule is (42.2 ± 0.8) Å^2^. The lattice transforms upon compression, and at 30 mN/m hexagonal packing of untilted chains is observed. The extrapolated transition pressure (23.1 mN/m) is lower than on the calcium containing subphase. Apparently, the presence of divalent calcium ions leads to a higher degree of dissociation compared to the monovalent sodium.

On the pH 9 subphase without calcium, a rectangular unit cell with chains tilted in the NNN direction is observed at low pressures. The tilt direction changes to NN at 30 mN/m, where the tilt amounts to (14.3 ± 1)° and the in-plane area per molecule is 42.4 Å^2^. The extrapolated tilting transition pressure is much higher (46.8 mN/m). On the pH 9 subphase with calcium, the oblique structure is seen at all pressures investigated. The tilt changes only marginally on compression (21 ± 1)°. Therefore, the structure is very rigid and does almost not respond on compression of the film. Furthermore, the extrapolated tilting transition pressure is an unrealistic value of 114 mN/m. The divalent calcium ions are able to bridge the highly charged phosphate groups and solidify the monolayer structure, in contrast to all the other systems. Calcium seems to be the key element to bridge the charged head groups leading to a very rigid arrangement of the molecules. It is interesting to note that calcium has this strong effect even in the presence of a 150-fold larger concentration of monovalent sodium ions.

**Figure 8:**
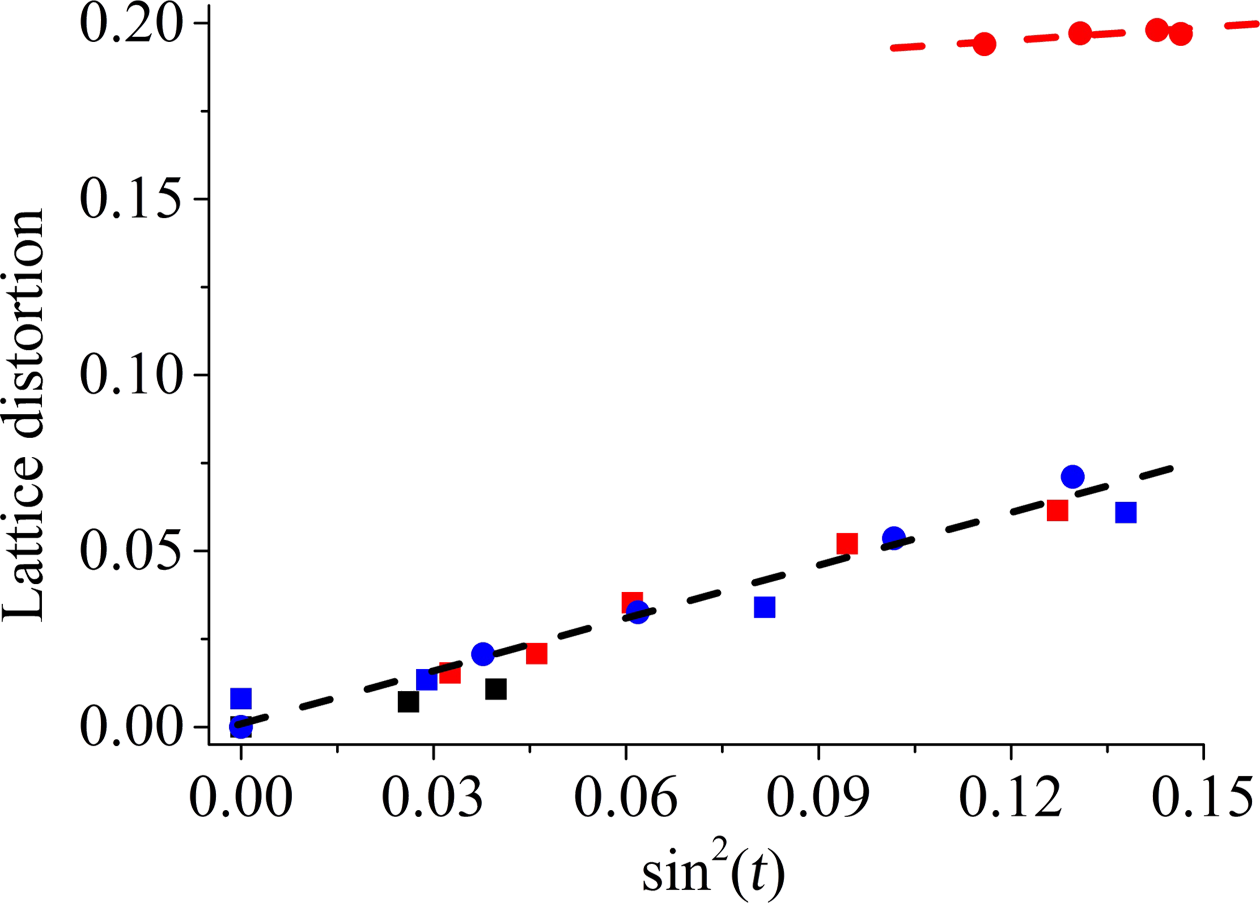
Lattice distortion as a function of sin^2^(*t*) of C1P monolayers at 20 °C on (red circle) pH 9 with calcium; (red square) pH 9 without calcium; (blue circle) pH 4 with calcium; (blue square) pH 4 without calcium; and (black square) on water.

Plotting the lattice distortion as a function of the sinus square of the tilt (Figure 8) according to a modified Landau theory usually shows a linear dependence [17]. An extrapolated zero distortion at zero tilt indicates that the lattice distortion is caused only by the tilt of the molecules [20]. However, this is not the case for the monolayer on the pH 9 subphase with calcium, since the distortion remains more or less constant at 0.18. The cross-sectional area is not extraordinarily small ((19.7 ± 0.04) Å^2^ at 30 mN/m) but is clearly smaller compared to all the other systems. Even if the value is not yet the one observed for herringbone or pseudo-herringbone packing, which is usually the source for lattice distortion apart from the chain tilting, the packing has clearly a strong influence on the distortion. Strong interactions in the hydrophilic head group region mediated by the calcium ions bridge the negatively charged phosphate head groups and enhance the solid arrangement of the molecules to form a strongly condensed monolayer.

The differences in the tilt transition pressure are in agreement with all the other data. On the pH 4 subphase without calcium, the C1P monolayer presents a mixture of one-fold deprotonated and neutral molecules. The electrostatic repulsions between the slightly negatively charged head groups are responsible for the larger areas per molecule and larger tilt angles. Upon compression, the strong van der Waals forces between the acyl chains and the screening effect of sodium ions surpass the electrostatic repulsions allowing C1P to transform into the untilted state. On the pH 4 subphase with calcium, the head groups interacting with calcium ions obviously require a larger effective area. Therefore, a much higher lateral pressure is needed to observe the untilted state. However, calcium is not able to bridge the single-charged head groups in an effective way as observed for the system on the pH 9 subphase. In the case of the pH 9 subphase without calcium, the untilted state is reached at much higher lateral pressures due to stronger electrostatic interactions. The presence of calcium ions leads to a very rigid structure, which does not change upon compression.

In a study of the Chalfant group [21], the activation of cPLA_2_α by C1P was found to be related with the chain length, showing an increase in activity with increasing chain length. It is known for other lipids that the chain length has an influence on the structural properties of the lipids. For example, in saturated fatty acids an expanded phase is dominantly found up to C_12_ chains, and lipids with longer chains exhibit only condensed phases [17]. Therefore, comparing the data of Chalfant and co-workers with our results, we suggest that the activation of cPLA_2_α by C1P is connected with the rigidity of the C1P structure, which is enhanced in the presence of calcium ions.

## Conclusion

The results presented clearly show the influence of the protonation degree of C1P head groups on its interaction with calcium ions. At higher pH without calcium ions, the C1P monolayer is at low lateral pressures in a liquid-expanded phase (LE) due to repulsive forces between the negatively charged phosphate head groups. At pH 4, the C1P head groups are either one-fold deprotonated or neutral (most probably a mixture of protonation states). The strong van der Waals forces between the chains allow the system to be in a condensed state. When calcium is added to the subphase, at both pH values, only a condensed phase state is observed. At pH 4, the systems with and without calcium are quite similar. However, the untilted state on the subphase with calcium is reached only at higher lateral pressures compared to the system without calcium. This intriguing behaviour let us believe that calcium is able to stabilize the one-fold deprotonated molecules in the system, even though less effectively than in the presence of two negative charges in the head groups. Sodium does not seem to be able to neutralize the negative charges so efficiently. At pH 9, when the C1P is mostly deprotonated, the divalent calcium interacts strongly with the negatively charged phosphate groups and bridges the head groups, enhancing a solid-like arrangement of the molecules in a condensed phase. The solidification effect of the monolayer by calcium ions is clear since the same effect is not seen with the 150-fold larger concentration of sodium ions.

This work shows clearly the affinity of calcium for the phosphomonoester of C1P and its big influence on the phase state of the lipid, which is pH-dependent (competition between repulsive electrostatic and attractive van der Waals forces). This result supports the calcium-mediated interaction of C1P with the C2 domain of cPLa2α (effector protein).

Since C1P is involved in many biological functions, its interaction with other ions could also be of interest. Furthermore, the potential to form solid-like domains (rafts) will critically depend on the intracellular pH and the presence of divalent cations, therefore the information presented in this work will help to better understand some of these biological mechanisms.

## Experimental

### Materials

Ceramide-1-phosphate (*N*-hexadecanoyl-D-*erythro*-sphingosine-1-phosphate) was purchased from Matreya, LLC (PA, USA). EDTA (≥99.4%), NaCl (≥99.5%), NaOH (≥99.5%), CaCl_2_ (≥99%) and HCl were purchased from Sigma Aldrich GmbH (Taufkirchen, Germany). Chloroform (≥99.9%) and citric acid monohydrate (≥99.5%) were purchased from Carl Roth GmbH (Karlsruhe, Germany) and methanol (≥99.9%) from VWR Chemicals (Fontenay-sous-Bois, France). Sodium tetraborate decahydrate (≥99.5%) was purchased from Merck KGaA (Darmstadt, Germany). NaCl was heated to 600 °C to remove potential organic impurities. All other chemicals and salts were used without further purification. For monolayer experiments, Milli-Q Millipore water with a specific resistance of 18.2 MΩ·cm and pH approx. 6.2 was used.

### Monolayer experiments

For monolayer experiments, a stock solution with a concentration of around 1 mM of C1P was prepared in a chloroform/methanol/0.5 N HCl (2:0.9:0.1 v/v) mixture, and vortexed until the solution was completely limpid. This mixture of solvents has been proved previously to be the most adequate one to dissolve C1P [8]. A 10 mM citrate buffer solution was used as subphase for pH 4 and a 10 mM borax solution was used for pH 9 (pH adjusted with NaOH/HCl). NaCl in a concentration of 150 mM was added to mimic the physiological environment, and 1 mM EDTA to chelate traces of divalent cations in the buffer solution. In case of a subphase with calcium, the EDTA was replaced by 0.001 M CaCl_2_.

The stock solutions were drop-wise given onto the subphase using a 100 µL micro-syringe, and 10 min were allowed for the evaporation of the solvent. The pressure–area isotherms were recorded on a computer interfaced custom-made Langmuir trough. The trough is equipped with a Wilhelmy balance using a glass plate. The compression speed was 3 Å^2^·molecule^−1^·min^−1^. The temperature was kept constant at the desired value with an accuracy of ±0.1 °C.

### Infrared reflection–absorption spectroscopy (IRRAS)

Infrared reflection–absorption spectra were recorded on a Vertex 70 FTIR spectrometer (Bruker, Ettlingen, Germany). The setup includes a MCT detector cooled with liquid nitrogen and coupled to a film balance (R&K, Potsdam, Germany), placed in an enclosed container to allow for a constant water vapor atmosphere. A sample trough with two movable barriers and a reference trough (subphase without monolayer) allows for the fast recording of sample and reference spectra successively by a shuttle technique. The infrared beam is focused on the liquid surface by a set of mirrors. The angle of incidence normal to the surface can be varied by moveable arms. A KRS-5 wire grid polarizer is used to polarize the infrared radiation either in parallel (p) or perpendicular (s) direction. Reflectance–absorbance spectra were obtained by using –log(*R*/*R*_0_), with *R* being the reflectance of the film covered surface and *R*_0_ being the reflectance of the same subphase without the film. Each IRRAS experiment was started 1 h after spreading. FTIR spectra were collected with a scanning velocity of 20 kHz and a resolution of 8 cm^−1^, using 200 scans for s-polarized light and 400 scans for p-polarized light and saved in OPUS 6.0^®^. For data analysis, spectra obtained with s-polarized light at 40° angle of incidence were used. IRRA spectra were shifted to zero between 1950 cm^−1^ and 2250 cm^−1^. To determine the position of the asymmetric CH_2_-stretching vibration band, a non-linear fit using Lorentz function in Origin 9^®^ was used.

### Brewster angle microscopy (BAM)

The morphology of the monolayer was imaged with a Brewster angle microscope, BAM2plus from NanoFilm Technologie (Göttingen, Germany), equipped with a miniature film balance from NIMA Technology (Coventry, UK). The microscope was equipped with a frequency-doubled Nd:YAG laser (532 nm, ca. 50 mW), a polarizer, an analyzer, and a CCD camera. When p-polarized light is directed onto the pure air/water interface at the Brewster angle (approx. 53.1°), zero reflectivity is observed. When a monolayer is added, the light starts to be reflected due to the change of the refractive index of the surface layer. This change is registered by the CCD camera after passing the analyzer. BAM images of 355 × 470 µm^2^ were digitally recorded during compression of the monolayer. The lateral resolution is ca. 2 µm.

### Grazing incidence X-ray diffraction (GIXD)

Grazing incidence X-ray diffraction (GIXD) experiments were performed at the beamlines BW1 at DESY, Hamburg, Germany, and ID10 at the ERSF, Grenoble, France. The setup includes a Langmuir trough, equipped with one moveable barrier and a Wilhelmy surface tension sensor. The temperature was kept at 20 °C by a thermostat. During experiments, the trough was kept hermetically sealed and flushed with He.

At BW1 (DESY, Hamburg) the synchrotron beam was monochromated through a beryllium(002) crystal to a wavelength of 1.304 Å (energy: 9.5 keV). The incidence angle at the liquid surface was 0.11°, which is around 85% of the critical angle for total external reflection for water at this X-ray wavelength. A Mythen detector (Paul Scherrer Institute, Villigen, Switzerland) was rotated around the sample in order to detect the intensity of the diffracted beam as a function of the vertical scattering angle α_f_ and the horizontal scattering angle 2θ. A Soller collimator (JJ X-Ray, Denmark) was located between the sample and the detector.

At ID10 (ESRF, Grenoble) the synchrotron beam was monochromated by a germanium(111) crystal to a wavelength of 0.56 Å (energy: 22 keV). The incidence angle at the liquid surface was 0.033°, which is around 85% of the critical angle for total external reflection for water at this X-ray wavelength. A Mythen detector (Dectrys, Baden, Switzerland) was rotated around the sample in order to detect the intensity of the diffracted beam as a function of the vertical scattering angle α_f_ and the horizontal scattering angle 2θ. A Soller collimator (JJ X-Ray, Denmark) was located between the sample and the detector. The setup was already described elsewhere [22–23].

The accumulated position-resolved counts were corrected for polarization, effective area, and Lorentz factor. In the case of ID10 the contour plots of the corrected intensities were plotted as a function of the in-plane (*Q_xy_*) and out-of-plane (*Q_z_*) components of the scattering vector in PyMca and cuts representing the Bragg peaks of the in-plane and Bragg rods of the out-of-plane direction were determined. Model peaks (Lorentzian in the in-plane direction (*Q_xy_*) and Gaussian in the out-of-plane direction (*Q_z_*)) were fitted to the corrected intensities, and structural information, as the tilt, distortion, cross-sectional area (*A*_0_) and the in-plane lattice area of one chain (*A_xy_*) were extracted [16–18].

## Supporting Information

File 1 Full experimental data.
