# Supplementary material for: Influence of calcium on ceramide-1-phosphate monolayers
Source: Beilstein J Nanotechnol. 2016 Feb 12;7:236–45. doi: 10.3762/bjnano.7.22 (PMC4778505; doi:10.3762/bjnano.7.22)
Supplement: File 1 — Full experimental data. [file Beilstein_J_Nanotechnol-07-236-s001.pdf]

Supporting Information

for

Influence of calcium on ceramide-1-phosphate monolayers

Joana S. L. Oliveira*[1], Gerald Brezesinski[1], Alexandra Hill[2] and Arne Gericke[2]

Address: [1]Max Planck Institute of Colloids and Interfaces, Colloid Chemistry Department, Wissenschaftspark Potsdam-Golm, Am Mühlenberg 1, 14476 Potsdam, Germany and [2]Department of Biological Sciences, Kent State University, Kent, Ohio 44242, USA

Email: Joana Santos Lapa Oliveira* - joana.oliveira@mpikg.mpg.de

[*] Corresponding author

Full experimental data

1 Thermodynamic analysis of C1P on pH 9 subphase without calcium

At pH 9 without calcium, C1P undergoes a first-order phase transition from the LE to the LC phase, which can be seen to start at 15 °C. Such transition can be related to the change in area by the two-dimensional Clausius–Clapeyron equation: $\Delta H_c/T = \Delta S_{Tr} = (\delta\pi_c/\delta T)\cdot\Delta A$, where ΔH_c and ΔS_{Tr} are the transition enthalpy and entropy (per molecule), respectively, between two coexisting phases, π_c and T are the surface pressure and temperature, respectively, at which the phase transition occurs and ΔA is the difference in area per molecule between the LE area and the LC area at the transition surface pressure. The transition enthalpy for such system can then be calculated by $\Delta Q = T\Delta S$. The values obtained for this system are presented in Table S1.

Table S1: Thermodynamic parameters of the analysed system.

T (K)	π_c (mN/m)	A_{LC} (Å^2/molecule)	A_{LE}	$A_{LC} - A_{LE}$	ΔS_{Tr} (J/mol·K)	ΔQ_{Tr} (kJ/mol)
293.15	6.4	48.9	70.2	−21.3	−148.91	−43.65
301.15	14.1	46.8	58.8	−12	−83.89	−25.26
303.15	18.8	45.6	55	−9.4	−65.71	−19.92

The extrapolation of the linear fit of $\pi_c = f(T)$ towards $\pi_c = 0$ leads to 14.7 °C. On the other hand, the critical temperature at which it is no longer possible to obtain a LC phase can be determined by extrapolation of the linear fit of $\Delta Q_{Tr} = f(T)$ towards $\Delta Q_{Tr} = 0$, which leads to 38.1 °C.

2 Surface pressure–area isotherm of C1P

In Figure S1 the surface pressure–area isotherm of C1P on water at 20°C is presented.

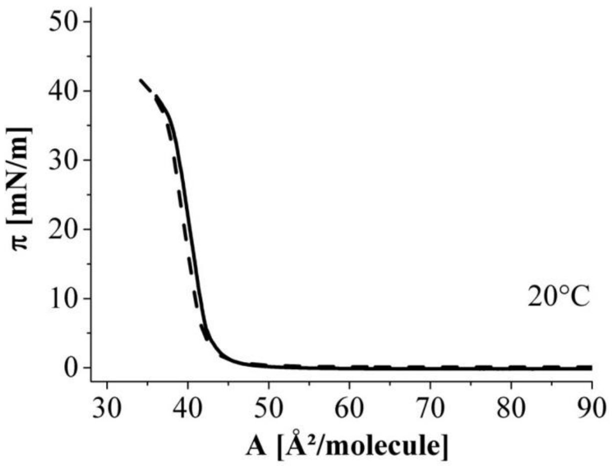

Figure S1: Surface pressure–area isotherm of C1P on water at 20°C.

3 GIXD measurements

In Tables S2–S6, the GIXD peak positions (Q_{xy} and Q_z) with tilt (*t*), distortion (*d*), cross-sectional area (A_0), and in-plane lattice area of a single chain (Axy) are given for pH 9 and pH 4 with and without calcium and on water.

Table S2: GIXD measurements on water at 20 °C.

π (mN/m)	Q_{xy}^1 (Å^{-1})	Q_z^1 (Å^{-1})	Q_{xy}^2 (Å^{-1})	Q_z^2 (Å^{-1})	t (°)	d	A_0 (Å^2)	A_{xy} (Å^2)
0	1.48	0.30	1.50	0.15	11.5 ± 1	0.0107	20.1 ± 0.4	20.5 ± 0.4
1	1.49	0.24	1.50	0.12	9.3 ± 1	0.0071	20.2 ± 0.4	20.4 ± 0.4
5	1.50	0	1.50	0.06	2.6 ± 1	0	20.2 ± 0.4	20.2 ± 0.4
10	1.51	0	—	—	0	0	20.1 ± 0.4	20.1 ± 0.4
20	1.52	0	—	—	0	0	19.8 ± 0.4	19.8 ± 0.4

Table S3: GIXD measurements at pH 9 and 20 °C (borax buffer with 150 mM NaCl and 1 mM $CaCl_2$).

π (mN/m)	Q_{xy}^1 (Å^{-1})	Q_z^1 (Å^{-1})	Q_{xy}^2 (Å^{-1})	Q_z^2 (Å^{-1})	Q_{xy}^3 (Å^{-1})	Q_z^3 (Å^{-1})	t (°)	d	A_0 (Å^2)	A_{xy} (Å^2)
5	1.34	0.51	1.45	0.41	1.59	0.10	22.5 ± 1	0.197	20.1 ± 0.4	21.7 ± 0.4
10	1.35	0.51	1.45	0.40	1.60	0.10	22.2 ± 1	0.198	20.0 ± 0.4	21.6 ± 0.4
20	1.36	0.49	1.47	0.37	1.61	0.09	21.2 ± 1	0.197	19.8 ± 0.4	21.3 ± 0.4
30	1.36	0.47	1.48	0.34	1.61	0.09	19.9 ± 1	0.194	19.7 ± 0.4	21.0 ± 0.4

Table S4: GIXD measurements at pH 9 and 20 °C (borax buffer with 150 mM NaCl and 1 mM EDTA).

π (mN/m)	Q_{xy}^1 (Å^{-1})	Q_z^1 (Å^{-1})	Q_{xy}^2 (Å^{-1})	Q_z^2 (Å^{-1})	t (°)	d	A_0 (Å^2)	A_{xy} (Å^2)
15	1.40	0.53	1.46	0.27	20.9 ± 1	0.0615	20.5 ± 0.4	22.0 ± 0.4
20	1.41	0.46	1.47	0.23	17.9 ± 1	0.0521	20.6 ± 0.4	21.7 ± 0.4
30	1.44	0.37	1.48	0.18	14.3 ± 1	0.0353	20.5 ± 0.4	21.2 ± 0.4
35	1.47	0.28	1.49	0	12.4 ± 1	0.0208	20.4 ± 0.4	20.9 ± 0.4
40	1.48	0.24	1.50	0	10.4 ± 1	0.0153	20.3 ± 0.4	20.7 ± 0.4

Table S5: GIXD measurements at pH 4 and 20 °C (citric buffer with 150 mM NaCl and 1 mM EDTA).

π (mN/m)	Q_{xy}^1 (Å^{-1})	Q_z^1 (Å^{-1})	Q_{xy}^2 (Å^{-1})	Q_z^2 (Å^{-1})	Q_{xy}^3 (Å^{-1})	Q_z^3 (Å^{-1})	t (°)	d	A_0 (Å^2)	A_{xy} (Å^2)
5	1.42	0.55	1.44	0.39	1.49	0.16	21.8 ± 1	0.0609	20.2 ± 0.4	21.7 ± 0.4
10	1.45	0.42	1.47	0.29	1.49	0.12	16.6 ± 1	0.0340	20.2 ± 0.4	21.1 ± 0.4
20	—	—	1.48	0.22	1.50	0	9.8 ± 1	0.0135	20.4 ± 0.4	20.7 ± 0.4
30	—	—	—	—	1.50	0	0	0	20.2 ± 0.4	20.2 ± 0.4

Table S6: GIXD measurement at pH 4 and 20 °C (citric buffer with 150 mM NaCl and 1 mM $CaCl_2$).

π (mN/m)	Q_{xy}^1 (Å^{-1})	Q_z^1 (Å^{-1})	Q_{xy}^2 (Å^{-1})	Q_z^2 (Å^{-1})	Q_{xy}^3 (Å^{-1})	Q_z^3 (Å^{-1})	t (°)	d	A_0 (Å^2)	A_{xy} (Å^2)
2	1.41	0.52	1.44	0.37	1.49	0.15	21.1±1	0.0711	20.4±0.4	21.9±0.4
10	1.43	0.46	1.45	0.35	1.50	0.10	18.6±1	0.0536	20.4±0.4	21.5±0.4
20	—	—	1.47	0.32	1.50	0	14.4±1	0.0356	20.2±0.4	20.9±0.4
30	—	—	1.48	0.25	1.50	0	11.2±1	0.0207	20.2±0.4	20.6±0.4
40	—	—	—	—	1.51	0	0	0	20.1±0.4	20.1±0.4
